# Development and evaluation of a support program for prostate cancer survivors in Alaska

**DOI:** 10.3402/ijch.v74.28605

**Published:** 2015-11-25

**Authors:** Stacy Kelley, Christine DeCourtney, Julia Thorsness

**Affiliations:** 1 Community Health Services, Alaska Native Tribal Health Consortium, Anchorage, AK, USA; 2 State of Alaska Department of Health and Social Services, Anchorage, AK, USA

**Keywords:** survivorship, prostate cancer, social support, program evaluation, disparities

## Abstract

**Background:**

Prostate cancer survivors in Alaska and elsewhere have unmet support needs. The Men's Prostate Cancer Survivorship Retreat, or “men's retreat,” was developed targeting Alaska Native and non-Native men who were survivors of prostate cancer. The program brought together survivors in a supportive environment to discuss and share their experiences.

**Objective:**

Despite the proven effectiveness of support groups for improving quality of life for cancer patients, men typically do not participate in formal support groups. This descriptive study was conducted to explore the needs of Alaska Native and non-Native prostate cancer survivors and assess satisfaction and acceptability of a men's cancer survivorship retreat in Alaska.

**Methods:**

Prostate cancer survivors (N=80) who attended men's retreats during 2009–2013 were asked to complete a retreat application and post-retreat evaluation. Comments regarding social support, helpful and valuable aspects of the retreat including overall satisfaction were reported.

**Results:**

A men's retreat with activities that engage men can be successful for prostate cancer survivors. Many men returned for successive retreats. After the retreat, 97% of the participants said they would continue with support activities.

**Conclusion:**

The men's retreat provides a valued opportunity for men to interact with other survivors and access information from health professionals. The results from this study highlight a successful model for social support and resources specific to male prostate cancer survivors.

During the last 25 years there has been a remarkable transformation of cancer from a fatal disease to one in which many individuals now receive effective treatments that result in long-term, disease-free survival (1). Despite this encouraging news about survival, research shows that a diagnosis of cancer often results in significant psychological distress and unsatisfactory quality of life (QOL), starting from the time of diagnosis and continuing throughout treatment and recovery (2).

An estimated 233,000 new cases of prostate cancer will have occurred in the United States in 2014 (3), and men who survive prostate cancer have unique chronic side effects compared with other cancer survivors. Their survivorship is often complicated by urinary, bowel, sexual and hormonal dysfunction, which challenge a man's physical and social well-being and life satisfaction (4). Furthermore, mental health is affected, with the incidence of depression and anxiety in prostate cancer survivors reported to be significantly higher than in the general population (5). Acute depression and anxiety may negatively influence treatment compliance (6), which in turn may result in increased hospitalizations (7) and worse overall functional QOL during and after treatment (8).

Social support, an indicator of social well-being, has received extensive attention in the context of QOL for cancer patients (9). Despite the proven effectiveness of support groups for improving QOL for cancer patients, men typically do not participate in formal support groups (10). Various barriers may explain the reasons why men typically do not participate. For example, men are less likely than women to seek help in general; older men are less likely to agree to psychological evaluation or to admit emotional distress; and clinicians underestimate the psychological morbidity of men with prostate cancer and don't refer patients to support groups (11). Furthermore, studies indicate that men with prostate cancer often lack awareness of support services, or they may simply feel they already have adequate support (12). Finally, it has been known for years that many men with prostate cancer may avoid disclosure about their illness as part of continuing a normal life (13, 14), while other male cancer survivors (prostate cancer survivors and survivors of other cancers) report participation in support groups to be stigmatizing (15).

Men who do participate in support groups are mostly retired and white, with an above-average education level (16). Men who participate in social support groups report satisfaction with the experience and have fewer somatic complaints than men who do not participate (17). Black men, however, are less likely than their white counterparts to attend support groups, (18), and the same is true for other ethnic minority groups (19).

A prerequisite for participation in support groups is the existence of such groups in formats that men perceive as meeting their needs (20). In Alaska, support-group services for men with prostate cancer are very limited. Indeed, prior to the intervention described in this report, the only face-to-face intervention for prostate cancer survivors was the nationally supported group Us Too, implemented on a limited scale at a local hospital. Other support services included only written resources and general cancer support groups not specific to men or to prostate cancer.

Recognizing the need and gap in services, the State of Alaska and Alaska Native Tribal Health Consortium (ANTHC) Comprehensive Cancer Control programs collaborated to develop a prostate cancer support program acceptable to Alaskan men. This paper reports on the development and outcomes of that program.

## Methodological approach

### Overall program design

In 2009, a planning team was convened to guide the development of a psychosocial support program for Alaskan prostate cancer survivors. Team members included representatives of local hospitals as well as urology and radiation oncology clinics, including clinic nurses, nurse case managers and social workers. Also included were representatives from the local chapter of Us Too, State of Alaska and Tribal Comprehensive Cancer Programs and tribal leaders from the Alaska Native community interested in prostate cancer survivorship issues.

It was decided that the best way to gain the interest of men was to provide the social support and clinical advice typical in standard support groups, in an Alaskan outdoor setting. By offering an outdoor activity in combination with access to clinical experts and no- or low-cost enrolment, we hoped to reduce barriers to participation.

The Men's Prostate Cancer Survivorship Retreat, or “men's retreat,” was thus developed for Alaska Native and non-Native men living in Anchorage, Alaska, and surrounding communities who had completed prostate cancer treatment. The weekend retreat, held at a fishing lodge, included evening discussions on cancer survivorship led by male clinical experts and trained support group leaders. A half-day guided activity of fly fishing, river rafting or hiking was offered as both an enticement to attend and a bonding activity for participants.

### Support elements

A urologist and radiation oncologist served as content experts for both one-on-one and group discussions. These 2 clinicians had extensive experience working with Alaska Native populations and were known to them. This familiarity was deemed important to encourage participation by Alaska Native men.

Trained Us Too group leaders were enlisted to facilitate formal group discussions and provide resources from Us Too. In addition, linkage with the Us Too program provided the opportunity for ongoing support after the retreat ended.

Funding for the event was secured from private urology clinic and hospital sponsorships. Additionally, small grant awards from the local prostate cancer awareness group and from state and tribal cancer programs were also secured. Full scholarships were available to cover expenses for men unable to pay the cost of the recreational portions of the retreat, which totalled about $500 per participant.

A series of 6 different retreats took place over a 5-year period (2009–2013). Each lasted 3 days. A schedule of activities is shown in Table I.

**Table I:** Summary schedule of retreat activities

Day 1 (evening)	Time allotted	Day 2 (full day)	Time allotted	Day 3 (half-day)	Time allotted
Meet and greet reception in the fishing lodge	1.5 hr	Group breakfast	1 hr	Group breakfast	1 hr
Welcome dinner	1.5 hr	Activity of choice: fly fishing, rafting, hiking	5 hr	Optional facilitated discussion or free time	3 hr
Formal introductions, including a summary of each survivor's cancer experience	1 hr	Group lunch Optional afternoon seminar/guest lecture	1 hr 2 hr	Evaluation Group lunch Participants depart	30 min 1 hr
Fireside chat facilitated by clinicians and Us Too prostate support group leaders	1 hr	Free time Group dinner Fireside chat facilitated by clinicians and Us Too prostate support group leaders – topics decided by the group as stated on survivor applications	3 hr 1.5 hr 3 hr		

### Participant enrolment

Retreat applicant criteria included a diagnosis of prostate cancer and completion of initial treatment. No restrictions were placed on how long the treatment was, nor on the length of time since initial treatment was completed. Patients in active treatment were not eligible to participate in the retreat because they may have had different concerns than men who had completed treatment, thus altering group discussions topics.

Eligible men enrolled through a first-come, first-serve application process. Promotional materials and applications were distributed at local urological clinics, radiation therapy clinics, hospitals, the local American Cancer Society (ACS) Resource Center and the existing Us Too support group Alaska listserv. Targeted outreach to minority men known to have had prostate cancer was conducted through provider and nurse case manager–directed efforts at the Alaska Native Medical Center (ANMC) Departments of Urology and Oncology. ANMC clinic social worker and nursing staff sent save-the-date postcards to patients and called them by telephone. Some participants were selected by their physician if they were perceived to have a lack of social support, low confidence to talk with their provider or to be struggling with chronic health issues that were related to their cancer treatment. The local Alaska Prostate Cancer Coalition hosted outreach materials and a downloadable application on their website. All applications were submitted to the ANTHC Comprehensive Cancer Planning Program. Eligible men were accepted until the maximum number of enrolees (up to 16 for each of the 6 retreats) was reached.

The application form included demographic information, treatment and time in survivorship, as well as any questions participants would like addressed at the retreat. Evening discussions were tailored to address questions submitted by survivors. The application form also requested information about what survivorship and support resources they had availed themselves of. Collection of this information, and all other aspects of the retreat and its evaluation, was approved by the Alaska Area Institutional Review Board and appropriate tribal agencies.

## Outcome evaluation

In addition to the demographic and cancer survivorship information gathered on the application, participants were also asked to complete a 17-question post-retreat survey that addressed overall satisfaction with the retreat, important aspects of the weekend retreat and social support developed as a result of the retreat. The post-retreat surveys were completed on the last day of the retreat weekend event.

### Retreat satisfaction

Participants were asked about their overall satisfaction with the retreat. This question was scored on a 5-point Likert scale, where a score of 1 indicated disappointment and was illustrated on the questionnaire as a sad face and a score of 5 indicated full satisfaction, illustrated with a happy smiling face. They were also asked if they would recommend the retreat to other prostate cancer survivors (*yes* or *no*).

### Important parts of the retreat

Participants were asked to score how helpful parts of the retreat were including meeting fellow retreat participants, the evening educational program/discussions, ability to discuss topics with clinical staff, having “free time” to relax with fellow retreat participants and information provided on local resources. Ratings were on a 5-point scale ranging from 1 (indicating *not helpful*) to 5 (indicating *very helpful*).

A second question asked participants to identify more helpful or important parts of the retreat from a list of choices including meeting other cancer survivors, the evening program talks, the resources made available, being able to ask questions, the activities and other. The participants were able to check as many of these options as they wanted.

### Social support

Men had been asked on their retreat application to identify support resources they had accessed previously. They were also asked on the post-retreat evaluation if they intended to participate in any support groups in the future (*yes* or *no* and the group in which they intended to participate). The cancer survivors were asked with whom they usually discuss their recovery and cancer experience (*nobody*, *spouse*, *close family and/or friends only*, *family and friends*, *anyone*, *doctor*, *support group* and *other*). Men were not specifically asked if they were married or single, but they were asked if they felt their spouses or close family members should be invited to attend the retreat (*yes* or *no* with area to provide a comment).

### Data analysis

Questionnaire responses from prostate cancer survivors who attended the men's retreat and who completed the application and post-retreat evaluation were included in the analysis. For men who attended more than 1 of the 6 retreats (22 men), the analysis only considered data collected from their first retreat application. All post-retreat evaluations were included in the analysis, regardless of whether men had attended multiple events. Both quantitative and qualitative data from the application and post-retreat evaluation were tabulated and interpreted by the researcher. For the quantitative analysis, basic descriptive statistics were also calculated and reported. For the qualitative analysis, a thematic analysis technique guided the analysis of data presented in Tables III and IV. Two independent researchers were asked to verify the accuracy of the qualitative data, and after discussion with them minor modifications were made.

## Results

### Participants and their pre-retreat questions

A total of 56 different prostate cancer survivors attended the 6 retreats throughout the 5-year study period. A total of 88 men's retreat evaluations were included in the evaluation, as several men (n=22; 25%) attended more than 1 retreat. A total of 34 (37%) attended only 1 retreat; 17 (19%) attended 2 retreats; and 5 (6%) attended 3 or more.

Most participants were white (63%) and 23% were Alaska Native/American Indians. Table II shows the demographic characteristics and cancer treatment status of the participants. Table III lists the most frequent questions/topics that men entered on their applications as topics they wanted the clinicians to discuss during the retreat.

**Table II:** Characteristics of participants in the prostate cancer retreat, 2009–2013

Participant demographics	N (%)
Age	N=56
50–59	8 (14)
60–69	27 (46)
70–79	20 (36)
80 +	2 (4)
Residence	N=56
Urban (Anchorage)	33 (57)
Rural (outside of Anchorage)	24 (43)
Ethnicity	N=56
White	36 (63)
Black	5 (8)
American Indian/Alaska Native	15 (26)
Hispanic/Latino	2 (3)
Survivorship	N=53^a^
< 1 year	22 (40)
2–4 years	13 (24)
5–9 years	10 (19)
10+ years	9 (17)
Cancer treatment type^b^	N=54^a^
Radiation	32 (59)
Surgery	29 (54)
Hormones	13 (24)
Chemotherapy	4 (7)
Other	3 (5)
More than 1 type of treatment	21 (39)

a Note: 56 individuals submitted unique applications, but only 53 provided survivorship information and 54 provided cancer treatment type;

b more than 1 treatment type was reported.

**Table III:** Five most frequent question asked by retreat participants on their enrolment application (2009–2013)

Topics/themes	N (%)	Exemplars
Incontinence	9 (16)	I would like to talk about bladder function problems and incontinence.
		I would like to talk about insurance companies that are refusing to pay for treatments my doctor is recommending.
Sexual function problems	17 (30)	I have severe pain after orgasm and my doctor is not familiar with that problem. I would like to discuss this with others.
Prevention	16 (30)	The role of exercise and prostate cancer prevention.
		Should prostate cancer survivors avoid fish oil? I would like to talk about nutrition for survivors.
Social support	21 (38)	Share my recovery story and how cancer affected me and my family.
		I look forward to the support of a shared experience with other men in a safe environment.
		I would like to hear about other men's treatments and how they are doing.
Reoccurrence	10 (16)	I would like to talk about my permanent side effects and continuing health problems.
		What options do I have if my cancer comes back?
		Possible spread from areas of my lymphatic system to “where next?”

A total of 76 participants completed a post-retreat evaluation. A total of 68 (89%) of participants scored their satisfaction with the retreat as a 5 (the highest level of satisfaction) and 8 (10%) scored it as a 4. Almost all (n=75; 99%) participants stated that they would recommend the retreat to fellow prostate cancer survivors. None of the 76 participants who completed a post-retreat evaluation reported being unsatisfied. Twelve did not complete an evaluation.

### Important parts of the retreat

Almost all (70%) of the participants felt that being able to meet other cancer survivors was the most important part of the retreat, followed by the evening program/discussions (59%) and being able to ask questions (57%). Fewer participants (25%) ranked written resources as important.

In response to questions about the value of education and social support aspects of the event, each category received a favourable score. The evening educational programs and information on local resources were both rated as 4.7 out of 5. Meeting fellow participants attending and having enough free time both received a mean score of 4.8 out of 5. Being able to discuss topics with clinical staff was the highest ranked, at 4.9 out of 5. Table IV presents additional comments from participants regarding the value of the retreat.

**Table IV:** Additional comments regarding value of the retreat

Topics/themes	Exemplars
Meeting other survivors	A significant emotional event that has helped me in many ways and has made me more open to helping others.
	To talk to and learn from others who had experienced prostate cancer diagnosis and various treatment options and the side effects of treatment.
	Meeting other survivors and hearing open testimony from them.
The evening discussions	Hearing confirmation that others have the same concerns and hope.
	Some of the discussion got a little repetitive hearing the same survivor story.
	It was sometimes difficult to hear, I am hard of hearing.
	I am not alone.
Being able to ask questions	Many of us were very open and candid in our discussions.
	Good to have the doctors available for questions and answers.
Unanticipated outcomes	Some of us meet once a week for coffee and sharing. I also email some men occasionally that live in Anchorage.
	The retreat gave me a great deal and allowed me to get more out of the meetings. I highly recommend a retreat to all survivors.
	I cannot tell you how important the retreat was in motivating those of us from [rural areas] to continue our [informal] local prostate support group's efforts.
	It was a nice addition to have the activities to develop relationships, but it was not necessary.

### Social support

Among the 56 who responded to the retreat application questions about whom they most often discuss their recovery with, 49% reported their spouse and 39% reported their physician. They also reported discussing their recovery with friends (32%), close family members (29%) and a support group (29%). A few (5%) reported having talked with no one about their cancer survivorship.

On the post-retreat evaluation, the majority of the men (57%) indicated that they would have liked the opportunity to invite their spouse or close family member to the retreat. However, it was noted in the comments by 3 men that they did not have a spouse or close family member to invite. It was also noted by 10 men that the richness of the evening discussions would not have been as open and honest if spouses or close family members were present.

Forty percent (n=56) of the men indicated on their retreat application that they had attended a formal cancer support group before the retreat. When asked on the post-retreat evaluation if they planned on attending any support group offerings in the future, 97% men indicated that they planned on joining an organized social support group like Us Too or becoming more involved with advocacy efforts involving cancer survivorship (such as the ACS, Alaska Prostate Cancer Coalition's Men's Run and LIVESTRONG Foundation).

## Discussion

### Clinical implications

Although the literature shows that men with prostate cancer tend not to participate in support activities, our study shows that a retreat with activities that engage men (not just discussions about cancer, but also bonding activities like fishing, hiking, etc.) can be successful in enlisting their participation. Indeed, many men returned for successive retreats, and after the retreat most said they would continue with support activities.

Through our quantitative and qualitative evaluation, we found the retreat format and content were acceptable to men and well received. Although the retreat activities were successful in enlisting the men to participate, the most valuable and important parts of the event were the ability to meet other survivors and having clinical staff available. The staff were able to address specific concerns relating to incontinence, erectile dysfunction, overall wellness and cancer reoccurrence. While men reported satisfaction with the events throughout the weekend, the facilitated evening discussions played an important part in allowing the men time to have concerns vetted with clinicians and to share their cancer experience with the group.

The overall findings suggest that developing resources for prostate cancer survivors that use informal settings facilitated by male health care providers and support group leaders may benefit men who typically would not attend a formal support group. Alaskan men tend to spend a great deal of time in the outdoors, so that type of setting was familiar to them. The retreat also provided a venue for men to hear and learn about ongoing resources and local advocacy efforts to become involved in.

There are limitations to the methods to consider when evaluating the results of our experience. The first is that the satisfaction survey was conducted on a self-selected group of men mostly from the Anchorage area. Therefore, the results from the survey may not be generalizable to the cancer survivorship population nationally and results may only be reflective of those who elected to participate in the men's retreat. The results were also limited to data gathered from retreat applications and post-retreat evaluations. Conducting a future follow-up evaluation (e.g. 1 year after the initial retreat) may provide additional useful information about social support relationships developed and QOL issues.

Another limitation includes participant self-report bias. The evaluation was a self-administered survey conducted at the end of the retreat and included questions regarding their personal views and their experiences with the retreat. Some men may have felt the need to complete a positive evaluation in the presence of the staff.

Despite those limitations, almost all men indicated that they would recommend the retreat to fellow Alaskan prostate cancer survivors. Many had specific comments regarding how the retreat has assisted them in their recovery efforts.

## Conclusion

This study explores the challenges and outcomes of implementing a men's cancer retreat program. Findings from this study provide useful information about developing retreat offerings to better serve male cancer survivors.

Social workers and other healthcare professionals that support cancer survivors are in a unique position to influence the delivery of programs and services that affect the QOL of prostate cancer patients. Keenly aware of the side effects from treatment, oncology social workers are also in a position to help direct men into unique social support resources or help facilitate/coordinate similar offerings. Our program model can be replicated and offered in other areas of the world that have natural/outdoor settings. Findings from this study could be extended and replicated. The program could be considered for pilot research for other specific cancers, including colorectal cancer and cancers of the neck and head as these diseases also cause long-term and chronic side effects, or for patients who suffer from other non-cancer, chronic diseases.

The results from this study highlight a successful model for social support and resources specific to male prostate cancer survivors in areas of Alaska. Although future research and program evaluation is warranted to further understand survivorship, long-term support and resource needs, this first step of a small program has been able to fill a need within the community.
